# Simultaneous pacing from two branches of coronary sinus in a patient with prosthetic tricuspid valve and complete heart block

**DOI:** 10.1186/s12872-020-01373-9

**Published:** 2020-02-10

**Authors:** Mustafa Yolcu

**Affiliations:** 1 Medicine Faculty, Department of Cardiology, Yeni Yuzyil Universty, İstanbul, Turkey; 2 Yeni Yüzyıl Üniversitesi Tıp Fakültesi Özel Gaziosmanpaşa Hastanesi merkez, mahallesi çukurçeşme caddesi no: 51, Gaziosmanpaşa/İstanbul/, Türkiye

**Keywords:** Complete heart block, Permanent pacemaker, Prosthetic tricuspid valve

## Abstract

**Background:**

Complete heart blocks underwent to permanent pacemaker placement are a common complication of tricuspid valve replacement (TVR). If indicated, endocardial placement of a right ventricular (RV) lead is precluded in the presence of mechanical TVR.

**Case presentation:**

A 20-year-old female patient firstly underwent metallic prosthetic valve operation with tricuspid valve endocarditis in 2014. Three years after the operation, echocardiography revealed dysfunction of the prosthetic valve thus reoperation was decided. In the second operation, the patient underwent a bioprosthesis valve and AV complete block developed in the postoperative period. Left ventricular ejection fraction (EF) was 45% was found on echocardiography. Pacemaker dependence of the patient, it was aimed to place two electrodes into the left ventricle. Electrodes were placed the target two branches in coronary sinus (CS) and right atrium. Univentricular bifocal pacing was enabled to work.

**Conclusion:**

Electrode placement in the CS is a very good alternative to epicardial surgical lead placement in cases where endocardial lead placement from the right atrium to the RV is contraindicated. In patients with lower left ventricular EF who will be pacemaker dependent, the insertion of two electrodes into the CS to prevent pacemaker is a safe and effective treatment.

## Background

Complete heart blocks underwent to permanent pacemaker placement are a common complication of tricuspid valve replacement (TVR) [1]. If indicated, endocardial placement of a right ventricular (RV) lead is precluded in the presence of mechanical TVR [1]. For the procedure of endocardial lead placement, tilting disk valve prosthesis is the absolute contraindication due to the risk of acute valve failure, damage to the lead, and death [2]. In the routine clinical practice transvenous RV endocardial lead placement is performed. However, the tendency to replace ventricular lead is through away via the epicardial approach or via the coronary sinus (CS) in most of these cases [3]. Epicardial pacemaker lead implantation which is performed via thoracotomy is usually associated with high threshold occurrence [4]. We present an univentricular bifocal pacemaker implantation in a patient who underwent tricuspid valve surgery twice and developed complete AV block after the second operation.

## Case presentation

**Fig. 1:**
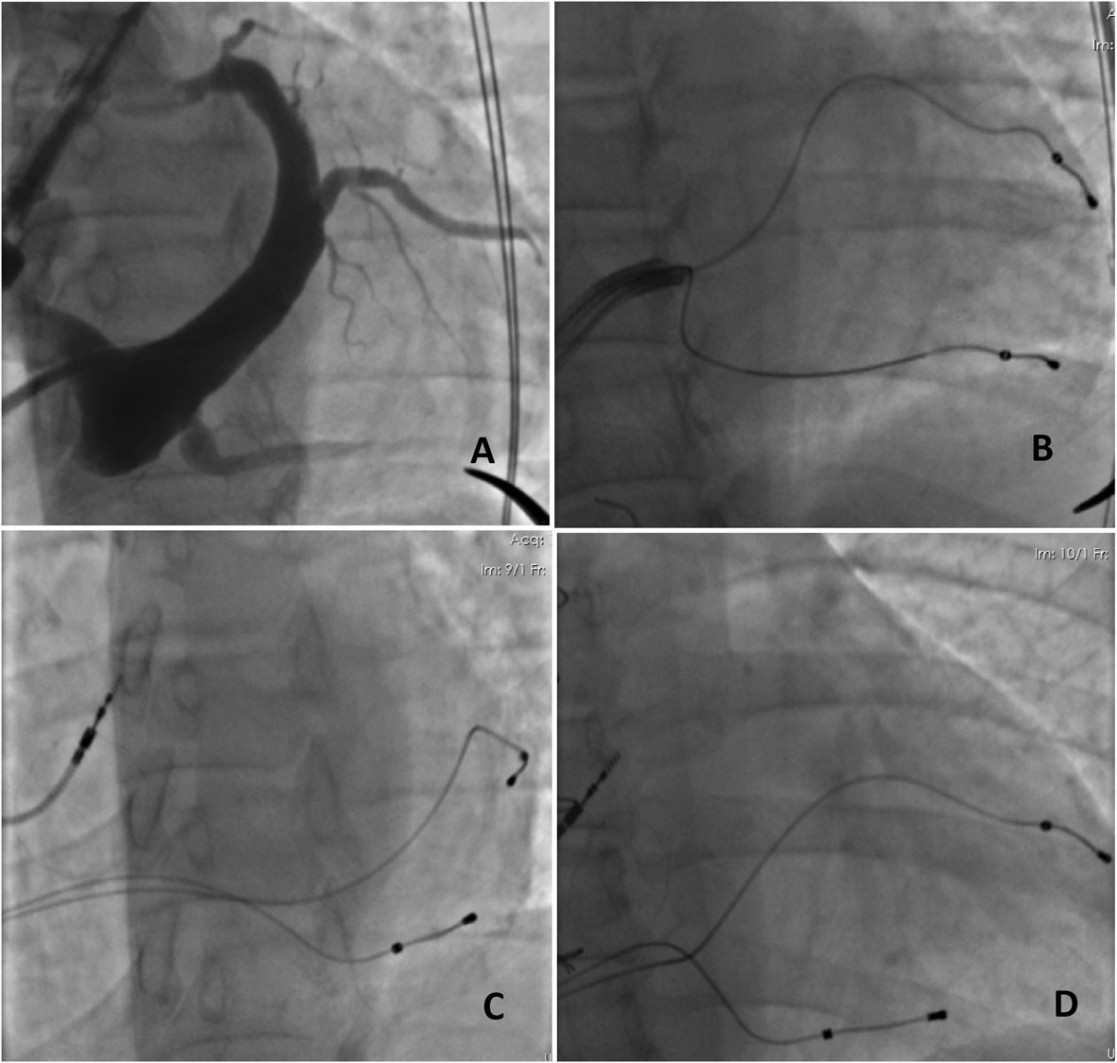
**a**. Coronary sinus angiography, **b**. Two sheats in coronary sinus, **c-d**. Right atrium and coronary sinus electrodes

**Fig. 2:**
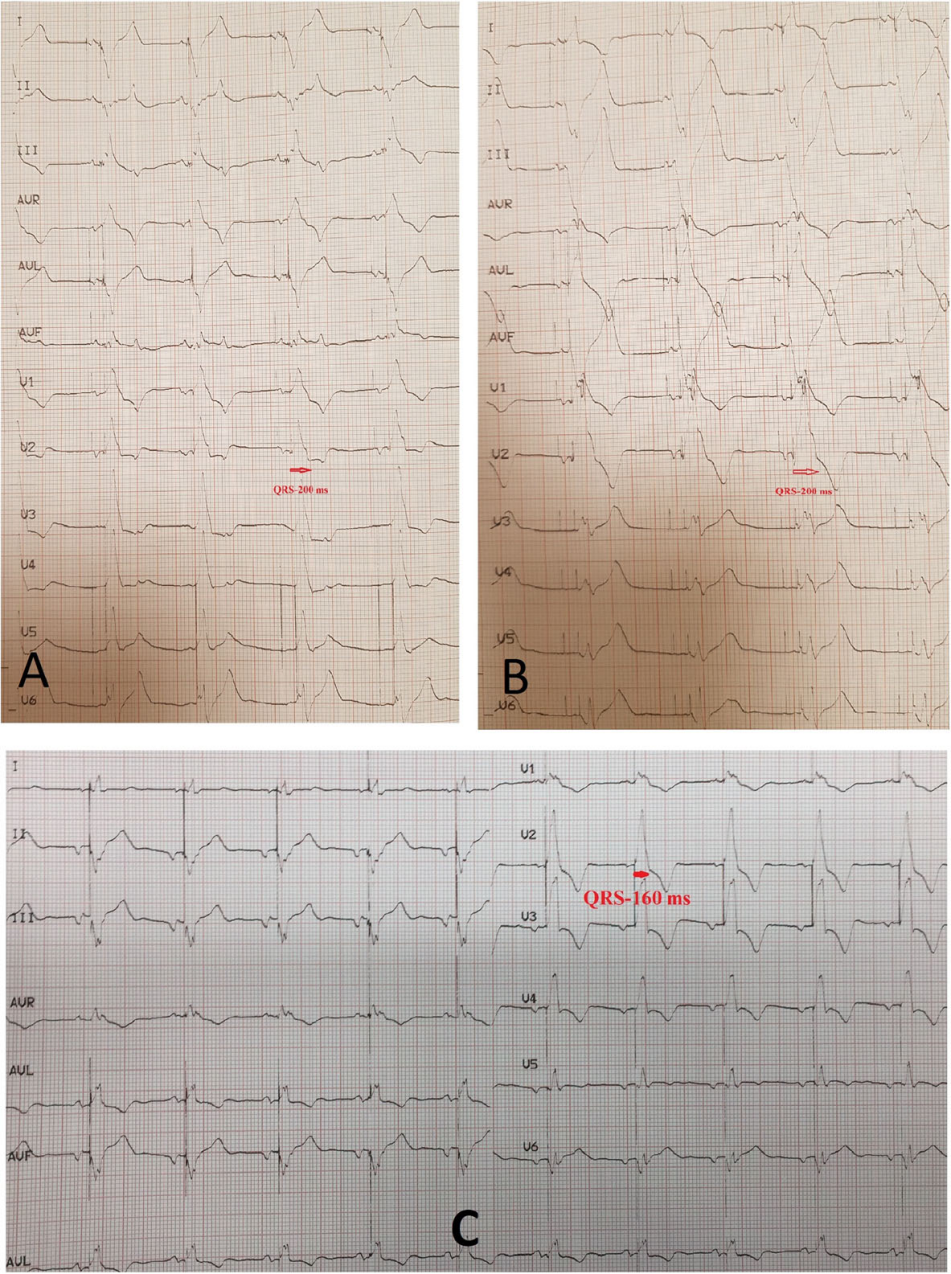
Electrocardiography, **a**. Coronary sinüs lateral branch pacing, **b**. Coronary sinüs middle cardiac vein pacing, **c**. Univentricular bifocal pacing

A 20-year-old female patient firstly underwent metallic prosthetic valve operation with tricuspid valve endocarditis in 2014. Three years after the operation, complaints of resistant ascites and dyspnea had been started. Electrocardiography (ECG) sinus rhythm revealed that the PR interval and QRS width were normal and there were no pathological ST-T wave changes. Echocardiography showed that the prosthetic tricuspid valve leaflets did not open and the maximum and mean gradients on the valve were 17 and 8 mmHg, respectively. Echocardiography revealed that prosthetic valve was dysfunctional thus reoperation was decided. In the second operation, a 27 mm St Jude Medical Epic porcine bioprosthetic valve was implanted in the patient and AV complete block developed in the postoperative period. Left ventricular ejection fraction (EF) was 45% and left ventricular dyssynchrony was found on echocardiography performed under transient pacemaker. Due to the low EF and pacemaker dependence of the patient, it was aimed to place two electrodes into the left ventricle and therefore reducing dyssynchrony. The patient underwent 3 left subclavian punctures and two simultaneous access systems were placed in the CS. In the CS angiography, lateral branch and middle cardiac veins were targeted (Fig. 1: a. Coronary sinus angiography, b. Two coronary sinus sheaths, c-d. Right atrium and coronary sinus electrodes). Electrodes were placed on the target two branches and right atrium. Threshold was below 1 V in both branches. When the CS was paced separately from the lateral branch and middle cardiac vein, the QRS duration was found as 200 ms. However, when the CS was paced simultaneously from the lateral branch and middle cardiac vein (univentricular bifocal pacing), the QRS duration was 160 ms (Fig. 2: Electrocardiography, a. Coronary sinus lateral branch pacing, b. Coronary sinus middle cardiac vein pacing, c. Univentricular bifocal pacing). Echocardiographic control at first month showed that EF was 50%. QRS width did not change during follow-up. Prosthetic valve function was normal in the 2-year follow-up of the patient, EF was around 55% and pacemaker measurements are within normal limits. Atrial pacing was not required in pacemaker control but there were intermittent episodes of paroxysmal atrial fibrillation, longest one being 46 h.

## Discussion and conclusions

The permanent pacemaker is applied in less than 1% rates after coronary artery bypass grafting and in between 3 to 6% rates after valve interventions according to the type of operation [2]. Transvenous right ventricular endocardial pacing lead might complicate to acute valvular dysfunction in such mechanical tricuspid prosthetic valves, therefore it shouldn’t be preferred [5]. In the past methods epicardial lead implantation was commonly performed via the anterolateral thoracotomy or sternotomy [5]. It was widely known that these highly invasive methods had many surgical risks [5]. Additionally, in these cases the need for reoperation is associated with significant risks, longer hospital stay time, and a high lead failure rate [5]. It was reported that, the epicardial leads cause high pacing thresholds on follow-up [6]. Transvenous left ventricular epicardial lead implantation through coronary vein is much less invasive than the surgical epicardial implantation [6].

In various case reports, usage of great and middle cardiac vein for permanent pacing in cases with tricuspid prosthesis was written. Anagnostopoulos et al. made the first successful left ventricular permanent pacing by using the great cardiac vein in 1970 [7]. Successful outcome was reported by Hansky et al. for left ventricular pacing in seven patients having TVR and one patient after tricuspid valve repair. There were no complications seen, also all devices had proper function [5].

Sirinivasan et al. placed defibrillated lead into CS in a patient with TVR and implantable cardioverter defibrillator indication for ventricular tachycardia [8]. The RV lead that was present before the TVR and that remained behind the prosthetic valve was also used to be worked as a biventricular pace [8]. The patient’s QRS decreased from 186 ms to 142 ms [8].

Vijayakumar et al. placed a VVI pacemaker via electrode inserted into the CS, on a patient with TVR due to a high threshold of epicardial lead which was placed during the operation [4].

Jokinen et al. intervened 136 tricuspid valves over 15 years and followed the patients for 7.9 ± 4.1 years [2]. The incidence of pacemaker placement was 21% (28 of 136 patients) after TV operation. This rate was apparently higher than the other valve interventions [2]. Before hospital discharge 54% of cases (15/28 patients) had pacemaker implantation. Whereas after hospital discharge, nearly half of them (13/28 patients) underwent implantation [2].

Electrode placement in the CS is a very good alternative to epicardial surgical lead placement in cases where endocardial lead placement from the right atrium to the RV is contraindicated. Different case reports suggest that a VVI or DDD pacemaker can be safely implanted via CS in the presence of a mechanical TVR. Our case had undergone tricuspid valve operation twice and right ventricular pacemaker implantation was contraindicated for her. As the patient had 45% left ventricular EF, it was thought to be she would be pace-dependent and this univentricular pace could further impair left ventricular performance. Two electrodes were placed in the CS. It was enabled to work as a univentricular bifocal pacemaker. QRS was measured as 160 ms on ECG after the procedure.

In patients with lower left ventricular EF who will be pacemaker dependent, the insertion of two electrodes into the CS to prevent pacemaker syndrome and prevention of dyssynchrony is a safe and effective treatment.

## Data Availability

The data used İn the literature review are available from the corresponding author on reasonable request.

## Abbreviations

CS: Coronary sinus
ECG: Electrocardiography
EF: Ejection fraction
RV: Right ventricle
TVR: Tricuspid valve replacement
